# Context-effect bias in capuchin monkeys (*Sapajus* spp.): exploring decoy influences in a value-based food choice task

**DOI:** 10.1007/s10071-022-01670-0

**Published:** 2022-09-20

**Authors:** Marco Marini, Chiara Boschetti, Serena Gastaldi, Elsa Addessi, Fabio Paglieri

**Affiliations:** 1 Department of Psychology, Sapienza University of Rome, Rome, Italy; 2 Department of Philosophy, Communication, and Performing Arts, Roma Tre University, Rome, Italy; 3 Unit of Cognitive Primatology, Istituto di Scienze e Tecnologie della Cognizione, Consiglio Nazionale delle Ricerche, Rome, Italy; 4 Goal-Oriented Agents Lab, Istituto di Scienze e Tecnologie della Cognizione, Consiglio Nazionale delle Ricerche, Rome, Italy

**Keywords:** Decoy effects, Capuchin monkeys, Comparative decision making, Context effects, Attraction effect, Repulsion effect

## Abstract

Decision making is known to be liable to several context effects. In particular, adding a seemingly irrelevant alternative (decoy) to a set of options can modify preferences: typically, by increasing choices towards whatever option clearly dominates the decoy (attraction effect), but occasionally also decreasing its appeal and generating a shift in the opposite direction (repulsion effect). Both types of decoy effects violate rational choice theory axioms and suggest dynamic processes of preference-formation, in which the value of each alternative is not determined a priori, but it is instead constructed by comparing options during the decision process. These effects are well documented, both in humans and in other species: e.g., amoebas, ants, honeybees, frogs, birds, cats, dogs. However, evidence of decoy effects in non-human primates remains surprisingly mixed. This study investigates decoy effects in capuchin monkeys (*Sapajus* spp.), manipulating time pressure across different conditions, to test whether such effects require time-consuming comparative processes among available alternatives. Whereas the time-dependent nature of decoy effects is a robust finding in the human literature, this is its first investigation in non-human animals. Our results show that capuchins exhibit an attraction effect with decoys targeting their preferred food, and that this effect disappears under time pressure; moreover, we observe preliminary evidence of a repulsion effect when decoys target instead the less-preferred food, possibly due to the larger distance between decoy and target in the attribute space. Taken together, these results provide valuable insight on the evolutionary roots of comparative decision making.

**Supplementary Information:**

The online version contains supplementary material available at 10.1007/s10071-022-01670-0.

## Introduction

During the last 30 years, a large corpus of empirical research has shown that rational choice theory and its utility maximization assumptions do not provide an adequate description of individuals’ decision-making behaviour (Huber et al. 1982; Tversky and Simonson 1993; Ariely 2009). In their choices, various animal species, including *Homo sapiens*, systematically violate several utility or fitness maximization principles. More specifically, according to the principle of independence of irrelevant alternatives and the principle of regularity, preferences should not be affected by the inclusion of other irrelevant options in the choice set (Von Neumann and Morgenstern 1947; Luce 1959). In contrast, it has been shown that both humans and non-human animals systematically change their preferences depending on the decisional context (Huber et al. 1982; Tversky and Simonson 1993; Rosati and Stevens 2009), being influenced by choice architecture, format of the options, order of presentation, and salience or similarity between the alternatives (for a review, see Frederick et al. 2014).

The asymmetric dominance effect (ADE, or attraction effect) is one of the clearest examples of how decision makers’ choices are affected by contextual features. It shows that adding a clearly inferior and seemingly irrelevant option (the decoy) to a binary-choice set strengthens the probability of choosing one of the first two alternatives (the target). Elicitation of ADE requires multi-attribute alternatives (at least two attributes for each option; e.g., quality and price) and a dominated structure of the decoy option (equally satisfying on one attribute but clearly inferior on the other attribute compared to the target alternative; for a review, see Lichters et al. 2015). To date, ADE in humans’ decision making has been observed in many real-life domains such as consumer choices (Huber et al. 1982; Heath and Chatterjee 1995), evaluation procedures (Slaughter et al. 1999), dating (Ariely 2009), gambling tasks (Cheng et al. 2012), political decisions (Herne 1997), intertemporal choices (Marini et al. 2020), medical (Schwartz and Chapman 1999) and legal decision-making (Kelman et al. 1996). Crucially, contexts effects have been observed both in perceptual tasks (e.g., selecting geometric figures with the largest area; Trueblood et al. 2013; Spektor et al. 2018) and in value-based choices (e.g., buying a smaller house near the city centre, or a bigger one in a quieter but less accessible area; Gluth et al. 2017; Mohr et al. 2017). The pervasiveness of ADE suggests that decoys can influence choice both at a low level of processing (i.e., salience, attention) and with respect to more complex cognitive operations (i.e., attribute comparisons, value integration), also considering that perceptual processing has a causal influence on cognitive deliberation (Krajbich et al. 2010; Hu and Yu 2014; Marini et al. 2020).

However, even though ADE is well established in a variety of domains, its pervasiveness outside of laboratory settings has been challenged (Frederick et al. 2014; Yang and Lynn 2014). Moreover, recent studies found that, under specific circumstances, the presence of an asymmetrically dominated decoy may also have a negative impact on the attractiveness of its target option, increasing instead choices for the competitor and thus eliciting a repulsion effect (Aaker 1991; Spektor et al. 2018; Liao et al. 2021; Evans et al. 2021). In their study, Liao and colleagues (2021) attributed the differential elicitation of these context effects to the distance between the target and the decoy options in the attribute space. Specifically, in valued-based choices, the attraction–repulsion effect would follow a concave U-shaped function reflecting the target-decoy distance. Another recent interpretation explains the repulsion effect through a tainting hypothesis (Simonson 2014). It suggests that an undesirable and unattractive option (the decoy) would contaminate its surrounding attribute space, making the target option less desirable and leading decision makers to prefer the other extreme, i.e. the competitor (Spektor et al. 2018; Kruis et al. 2020). The variety of possible impacts that a decoy can have on subjects’ decision, which include also compromise (Simonson 1989) and similarity effects (Tversky 1972), have prompted appeals to an overarching theoretical synthesis, capable of accounting for these various effects in a coherent manner (Spektor et al. 2021).

The fact that adding an irrelevant option to a choice set might, depending on the decision-making parameters, elicit either attraction or repulsion towards the target option does not undermine the challenge posed by these phenomena to traditional theories of rational choice: in both scenarios, a seemingly irrelevant alternative is demonstrated to systematically bias decision-making, highlighting the need for theoretical models capable of accounting for these context effects. Sequential sampling models (SSM) aim to face such challenge, describing decision making as a result of a comparative process between the available options (Turner et al. 2018). According to SSM (arising in the framework of the Multialternative Decision Field Theory, MDFT, Roe et al. 2001), the decision-making process is a dynamic procedure that evolves over time, in which decision makers develop subjective values of the options. These values fluctuate until reaching a decisional criterion (the subjective threshold of choice). During the choice, the options are systematically compared among them, and their subjective representation varies depending on the quantity and quality of other available alternatives (Wollschläger and Diederich 2020). Critically, SSM account for decoy effects both at a perceptual (low-) and at a cognitive (high-) level (Busemeyer et al. 2019) and have recently been found to provide excellent accounts of empirical data in both the consumer and perceptual domains (Turner et al. 2018). According to this comparative origin of attraction effects, the decoy option induces a negative preference state for itself and, simultaneously, a boosting effect for the dominant alternative (the target option; Hotaling et al. 2010). Moreover, extensions of this approach can also account for the manifestation of either attraction or repulsion effects as a result of the decoy presence, depending on subtle cues in the order of presentation of the alternatives (i.e., the sequential presentation of the same stimuli in a different order led to different context effects, Evans et al. 2021). Recently, empirical support for SSM came from studies that used eye tracking methodologies or time constraints. The former suggested that attributes are individually processed and alternatives are repeatedly compared on the relevant value (Noguchi and Stewart 2014; Marini et al. 2020). Moreover, coherently with the MDFT, which states that decoy effects take time to arise from the comparative process, it has been shown that, in a value-based task, limiting the decision time available resulted in a reduction (or an annulment) of the attraction effect (Pettibone 2012; Marini and Paglieri 2019; see also Król and Król 2019; Marini et al. 2020 for a thorough explanation of the role of the comparative process within the choice and its influence on response times). Findings on the influence of time pressure on repulsion effects are instead much scanter and less conclusive (e.g., Spektor et al. 2018 observed repulsion effects regardless of response times, even though they reported some fragile influence on effect size). More specifically, previous time-related studies assumed that limiting the time available (often using a customized time threshold) implies the decision maker not having enough time to carefully compare the options (in an attribute-wise approach; Noguchi and Stewart 2014). This reduced comparative process would attenuate the influence of the irrelevant dominated option on the subjective value of the target (Pettibone 2012; Marini and Paglieri 2019).

In an evolutionary perspective, attraction effects have also been documented among many non-human animals, both in terms of perceptual and value-based decision making. The experimental evidence ranges from unicellular slime moulds (Latty and Beekman 2011) to ants (Edwards and Pratt 2009), túngara frogs (Lea and Ryan 2015), honeybees (Shafir et al. 2002), birds (starlings: Bateson 2002; hummingbirds: Bateson et al. 2003; Morgan et al. 2012; gray jays: Shafir et al. 2002), cats (Scarpi 2011), and dogs (Jackson and Roberts 2021). Interestingly, there is at least one species, hummingbirds, in which both attraction (Bateson et al. 2003) and repulsion effects (Bateson et al. 2002) have been reported. On the one hand, decoy effects represent an evolutionary problem since, under certain circumstances, the decision maker would limit its own fitness (i.e., foraging ability; being sensitive to some contextual distortions that could improve or worsen its performance) by exhibiting suboptimal decisional behaviour (not leading to utility maximization; Cohen and Santos 2017). On the other hand, such evidence from animal studies testifies that contextual influences manipulated the choice behaviour well before the evolution of the typically human constructing preferences process, thus suggesting their evolutionarily ancient pervasive role in decision making. To date, the relevant literature on non-human animals seems to suggest that contextual influences on decision making are in place regardless of phylogenetically closeness to humans, but it is still unclear whether comparable or species-specific underlying mechanisms lead to these context-related systematic biases.

Surprisingly, findings on non-human primates, our closest relatives, are rather variable. In a first study, Parrish and colleagues (2015) tested the attraction effect in catarrhine primates, and specifically in rhesus macaques, using a perceptual computerized discrimination task. In this study, macaques had to choose the larger geometric figure in the choice set (figures varied in size and orientation), and decoy options were inserted in trinary trials targeting one of the previous alternatives (the vertical or horizontal one). Their results showed that macaques were influenced by the decoy addition, showing both a performance improvement when the decoy was dominated by the real larger rectangle and a decrease in correct figure detections when the decoy had the same orientation as the smaller baseline alternative. These findings seemed to suggest that context effects emerge quickly and early in the decisional processes and are not limited to complex decision making (for a similar result in humans, see Trueblood et al. 2013). However, in a following study using a computerized preference task (Parrish et al. 2018), which entails a more cognitively complex decisional situation, rhesus macaques did not show any substantial evidence of attraction effects.

Cohen and Santos (2017) obtained comparable negative results in a value-based task with capuchins monkeys, platyrrhine primates which are often studied as a comparative model for human cognition (Fragaszy et al. 2004). Indeed, capuchins have been observed to be affected by many typically human decisional biases (framing effect, Lakshminarayanan et al. 2011; loss aversion, Chen et al. 2006; endowment effect, Lakshminarayanan et al. 2008; inequity aversion, Brosnan and de Waal 2003). However, despite these analogies, in Cohen and Santos’s study (2017), capuchins did not show any preference reversal effect when choosing among food rewards in the presence of a decoy (for a similar negative finding in great apes, see Sanchez-Amaro et al. 2019). A problem common to these studies is that in most cases monkeys’ preferences were not accurately measured before the decoy introduction. Crucially, in the attraction effect literature on humans, it is well known that a dominated option is more likely to shift the decision maker preferences when alternatives are near to their indifference point (Huber et al. 2014). Vice versa, in the case of a strong a priori preference, the decoy addition, although affecting subjective values, may not be powerful enough to elicit a behavioural preference reversal.

A preliminary attempt to carefully measure capuchins’ preferences in an ADE food preference paradigm was recently made by Watzek and Brosnan (2020). In their study, the decoy trinary conditions (the ternary conditions in which monkeys faced choices among three different alternatives) were built after carefully assessing each monkey’s indifference point between two options, in order to maximize the likelihood of eliciting decoy effects. However, even if this study found an overvaluation of the preferred food when a decoy option was present (capuchins overvalued the target food when an inferior option was available, and they were two times more likely to choose it), this preliminary exploration did not comprehensively examine the direction of the attraction effect (i.e., only decoys targeting the preferred food were used, without testing decoys targeting the non-preferred food), and it did not investigate the underlying choice mechanisms (i.e., time pressure was not manipulated, to verify the role of comparative processes in the elicitation of the effect).

To date, it is still difficult to draw coherent conclusions on the occurrence of decoy effects in monkeys, given the scarcity of available reports, several methodological differences and inconsistencies in the results. Hence, the present study aims to investigate decoy effects in capuchins monkeys in a value-based food choice task. As in Watzek and Brosnan (2020), we carefully assessed monkeys’ preferences to implement trinary choices involving a decoy based on each monkey’s individual preference in binary-choice tasks. Moreover, we assessed the direction of the decoy effect, by testing how the presence of a decoy targeting either the favourite or the less favourite food options altered capuchins’ choices. Finally, we tested MDFT assumptions by administering two decoy conditions, with and without time pressure, thus shedding light on the underlying cognitive mechanisms of context effects in a non-human primate species. We assumed the following hypotheses:

*H1 Decoy effect*: we expected an increase in the preferences for the food alternatives targeted by an asymmetrically dominated decoy (attraction effect), similar to what has been observed by Watzek and Brosnan (2020). Moreover, we expected this effect to be symmetric both for favourite and less favourite targets of the decoy.

*H2 Time pressure***:** in line with SSM assumptions, we expected to observe an attraction effect only in the condition with no time pressure. In the time pressure condition, limiting the comparative process of the alternatives would reduce (or annul) the decoy effect.

## Methods

### Ethical note

All experimental procedures were approved by the Italian Ministry of Health (337/2017-PR to EA) and performed in full accordance with the Directive 2010/63/EU on the protection of animals used for scientific purposes.

### Subjects

We tested 14 capuchin monkeys (7 females, average age 25 years, range 11–35), belonging to four groups, hosted at the Primate Center of the Institute of Cognitive Sciences and Technologies of the National Research Council of Italy (ISTC-CNR), in Rome. Each capuchin group was housed in indoor–outdoor compartments. The outdoor compartments measured 65.4–139.5 m^3^, depending on group size, and the two indoor compartments measured a total of 25.4 m^3^. All compartments were furnished with wooden perches, tree trunks, and branches. Animals were never food deprived, and they received their main meal (a variety of cereals, monkey chow—Altromin-A, A. Rieper S.p.A., Vandoies, Italy—seasonal fruit and vegetables) in the afternoon, after testing was completed. Water was available ad libitum.

### General procedure

Capuchins were tested individually in testing compartments (each measuring 60 × 75 × 75 cm), attached to the indoor compartment. Testing occurred between 09:30 a.m. and 12:00 p.m., 5 days a week. Separation for individual testing was achieved by first splitting the group into smaller units by means of sliding doors and then allowing the subjects to enter the indoor compartment. Then, a single subject was allowed in the testing compartments through a sliding door. After each daily session, capuchins returned to their social group.

The study included the following phases: Preliminary food preference, Pre-test I, Baseline, Pretest II and Decoy, presented in a consecutive order. In all phases, capuchins faced either binary or trinary choices (for details see below). Choices were considered valid when the subject grasped only one food option; if the subject grasped two options at the same time, the trial was considered not valid and was immediately repeated. In all phases there were two experimenters: experimenter 1 placed the food options on the tray and operated the apparatus; experimenter 2 operated the stopwatch, scored the data, and—at the beginning of each trial (with the only exception of the Preliminary food preference phase)—hid the positioning of the food options from the animal’s sight inserting a black vertical panel between the experimenter 1 and the experimental subject and removed it as soon as the trial started. There was an intertrial interval of 10 s, starting when the animal put the last piece of food into the mouth. All experimental phases were video recorded with a Canon Legria HF R806. The study lasted 4 months, from February to June 2021.

### Experimental phases

#### Preliminary food preference phase

The goal of this phase was to identify for each subject a high-preferred familiar food (food A) and a less-preferred familiar food (food B), where the food A was chosen over the food B in 65–85% of the trials in two consecutive sessions. Each daily session consisted of 20 binary-choice trials (1A vs. 1B) presented in a pseudo-random order, counterbalancing right-left presentation. When a subject met the criterion in the first session, we carried out a second session with the same food pair on the following day. Otherwise, we chose a novel food pair for that subject until it met the criterion. Food was cut into pieces of approximately constant size and weight (dried plum, black olives: 0.25 g; raisins: 0.20 g; dried pineapple: 0.30 g; sunflower seed: 0.06 g; Rice Krispies: 0.02 g; Cheerios: 0.10 g); each piece was weighed on a digital scale (Gibertini Europe 1700; 0.1 g accuracy).

Each option consisted of one piece of food. We presented the options by means of a Plexiglas tray (27 × 40 cm). The options were placed in two small cavities of the tray, each positioned at 5.2 cm from the frontal edge of the tray and at 8 cm from a central panel that divided the tray into two equally sized portions. After the subject entered the testing compartment and positioned in its centre, the experimenter pushed the cart on which the Plexiglas tray was placed toward the compartment and then moved the Plexiglas tray within the subject’s reach, so that the subject could grasp one of the options.

#### Pre-test I phase

The goal of this phase was to ensure that the food amounts used in the subsequent phases were adequate to capture a shift of preferences between food A and food B. Each session consisted of 18 binary-choice trials between different food combinations (2A vs. 1B or 2A vs. 8B, 9 trials for each combination) presented in a pseudo-random order counterbalancing right-left presentation. We presented the options by means of a black tray (38 × 45 cm), placing them on two out of three yellow rectangles (7.5 × 5.5 cm), each positioned at 3.5 cm from the frontal edge of the tray and at 7.5 cm from each other. As in the Preliminary food preference phase, after the subject entered the testing compartment and positioned in its centre, the experimenter pushed the cart on which the black tray was placed toward the compartment and then moved the black tray within the subject’s reach. Monkeys completed Pre-test I when they chose 2A over 1B and 8B over 2A in at least seven out of nine trials, respectively, in two consecutive sessions.

#### Baseline phase

The goal of this phase was to identify, for each subject, two amounts of food B such that (i) the amount L (lower bound) was the largest quantity of food B that the monkey chose less than 50% of the time over food A (i.e., the largest quantity of the non-favourite food B chosen immediately before the shift in preferences from food A to food B) and (ii) the amount U (upper bound) was the smallest quantity of food B that the monkey chose more than 50% of the time over food A (i.e., the smallest quantity of the non-favourite food B chosen immediately after the shift in preferences from food A to food B). Each session consisted of 20 binary-choice trials between 2 units of food A and variable amounts of food B. In the same session, there were five type of trials, presented four time each in a pseudo-random order, counterbalancing right-left presentation of the options: 2A vs. 1B, 2A vs. 2B, 2A vs. 4B, 2A vs. 6B, and 2A vs. 8B. We presented the food options on the same tray used in the Pre-test I phase. Each subject completed five baseline sessions.

Only for the trials including the options corresponding to the lower bound and to the upper bound, from video clips we scored frame-by-frame RTs (by means of the Elan software, 6.0 version), since when the experimenter started moving the cart toward the monkey to when the monkey touched one of the two options. The RTs were subsequently used to calculate, for each subject, the time pressure applied in the Decoy phase.

#### Pre-test II phase

The goal of this phase was to ensure that capuchins perceived decoys as asymmetrical dominated by their targets. Decoys always consisted of the same type of food as their target, but half the amount. Each session consisted of 27 binary-choice trials between three types of options: 2A vs. 1A, L/2B vs. LB, U/2B vs. UB (9 trials each, presented in a pseudo-random order, counterbalancing right-left presentation). We presented the food options on the same tray used in the Pre-test I and in the Baseline phases. Monkeys completed Pre-test II when they chose the target over the decoy in at least seven out of nine trials in two consecutive sessions.

#### Decoy phase

In this phase, capuchins faced four types of trinary choices: (i) decoy targeting preferred food A when it was the preferred option in the baseline condition (i.e., 2A vs. 1A vs. LB); (ii) decoy targeting preferred food A when it was the non-preferred option in the baseline condition (2A vs. 1A vs. UB); (iii) decoy targeting non-preferred food B when it was the non-preferred option in the baseline condition (2A vs. L/2B vs. LB); iv) decoy targeting non-preferred food B when it was the preferred option in the baseline condition (2A vs. U/2B vs. UB). To sum up, we had a 2 timing (free and time pressure condition) × 2 bounds (lower and upper bounds) × 2 decoy (targeting the preferred/non-preferred food) design. In the decoy phase, we investigated the effect of an asymmetrically dominated decoy when it targeted both the preferred and non-preferred food to (i) exclude any interference of other decisional biases (i.e., salience or fatigue biases); (ii) explore the effect of a different distance between target and decoy options in the attribute space.

There were two within-subject conditions, with and without time pressure. In the condition with time pressure, subjects could make their choice within a time window corresponding, for each monkey, to the 90% of its average RT scored in the Baseline phase. While experimenter 1 operated the cart with the tray, experimenter 2 scored each subject’s RT live using a stopwatch. As in the baseline, we scored RTs since when the experimenter started moving the cart toward the monkey to when the monkey touched one of the available options. If capuchins failed to choose one of the options within their time window, the experimenter did not provide any reward and the next trial began. In the condition without time pressure, subjects did not have any time limit to make their choice. The order of presentation of the two conditions was counterbalanced across subjects.

Each of the two decoy conditions encompassed 10 20-trial sessions. In each session, we carried out five trials for each type of comparison, presented in a pseudo-random order, counterbalancing right-left presentation of the options but making sure that the decoy was always next to its target. The food choices were presented employing the same tray used in the Pre-test I, Baseline, and Pre-test II phases.

## Results

### Preliminary food preference phase

During the Preliminary Food Preference phase, for each subject we found a food pair where the food A was preferred over the food B in 65–85% of the trials in two consecutive sessions. All subjects showed rather consistent preferences between the two consecutive sessions (average 73.21%, range 70–80%). The food pairs chosen for each subject are reported in Table I in the Online Resources.

### Pre-test I phase

As reported in Table II (see Online Resources), all subjects preferred two pieces of the more preferred food A over one piece of the less-preferred food B and eight pieces of the less-preferred food B over two pieces of the more preferred food A. Nine subjects reached the criterion of choosing 2A over 1B and 8B over 2A in at least seven out of nine trials in two sessions, three subjects in three sessions, and two subjects in four sessions. Thus, the food amounts used were potentially adequate to capture a shift of preferences between food A and food in the following experimental phases.

### Baseline phase

Table III (in the Online Resources) reports the results of the Baseline phase, which aimed to find for each subject two amounts of food B, labelled L and B, such that (i) the amount L (lower bound) was the largest quantity of food B that the monkey chose less than 50% of the time over food A and (ii) the amount U (upper bound) was the smallest quantity of food B that the monkey chose more than 50% of the time over food A. In the Decoy phase, lower and upper bound values were used to set the amount of food B in the decoy options.

### Pre-test II phase

As reported in Table IV (see Online Resources), in the Pre-test II phase all subjects successfully chose the target option over the decoy option in at least seven out of nine trials.

### Decoy phase

Following Watzek and Brosnan (2020), we chose the food amounts to be used during the Decoy phase on the basis of capuchins’ preferences during the Baseline phase. This procedure allowed the administration of trinary decoy trials specifically customized for each subject. However, since capuchins had different baseline values, we could not analyse the whole set of trinary choices. That is because, in this study, decoys were built by halving target amounts: in the case of skewed baseline preferences, this led to violations of ADE assumptions, according to which decoy options must be manifestly inferior only to the target (otherwise, dominance is no longer asymmetric), whereas they must differ from the competitor alternative on both dimensions (for a review of these constraints, see Frederick et al. 2014). Consider, for example, this choice situation: 4B vs. 2B vs. 2A, in which we had a target option (4B), a competitor alternative (2A), and a decoy option that was built halving the target amount (2B). In this case, the decoy was dominated by the target option (4B) on the number of food units (4 vs. 2). However, this option was also dominated by the competitor alternative on the quality dimension (food A vs. B). Given this double dominance relationship, we had to exclude this trial as the decoy option 2B could affect subjective values of both the 4B option (because of a decreased amount of food units) and the 2A alternative (due to an inferior quality of the food). The trials that had to be excluded from the analysis, and the specific reasons for their exclusion, are listed in Table V (in the Online Resources): in general, the most frequent issue was that the decoy ended up being dominated by both target and competitor, and this affected mostly decoys targeting the less favourite food, whenever they were quantitatively equal or smaller than the competitor (favourite food).

Data for all trials are reported in Tables 1 and 2 (see also table VI in the Online Resources). We carried out conditional fixed-effects logistic regressions to assess whether subjects’ preferences for the 2A option (favourite food) were affected by session, trial number, phase (baseline vs. decoy), bound (lower bound vs. upper bound), time pressure (pressure vs. no pressure). The identity of the subject was included as a random effect. The significance of interaction effects was tested using the Wald test; non-significant interactions were dropped from the model and the analysis was run again. Preliminarily, we ran a mixed-effects linear regression with response times as the dependent variable: results shown that RTs were significantly longer in the no time pressure condition compared to the time pressure one (*z* = 41.29, coeff. = 813.74, *p* < 0.001), thereby confirming that the latter was indeed imposing relevant time constraints on the subject’s decision-making process. Subsequently, we ran two sets of analyses for the trials in which the decoy targeted the food A (pro-A trials) and in which the decoy targeted the food B (pro-B trials), respectively. Data were analysed with the software Stata IC (Version 14).

**Table 1:** Decoy phase, Time Pressure condition

Subject	Lower bound (LB)			Upper bound (UB)		
	Baseline	Decoy pro A	Decoy pro B	Baseline	Decoy pro A	Decoy pro B
	2A vs. LB	2A vs. 1A vs. LB	2A vs. L/2B vs. LB	2A vs. UB	2A vs. 1A vs. UB	2A vs. U/2B vs. UB
Cognac	0.90	0.82	0.90	**0.30**	**0.61**	0.56
Gal	0.80	0.84	0.92	**0.20**	**0.20**	0.24
Paprica	0.60	0.30	0.42	**0.00**	**0.02**	0.04
Patè	0.80	0.74	0.84	**0.25**	**0.12**	0.32
Penelope	0.65	0.46	0.38	**0.35**	**0.06**	0.02
Roberta	**0.65**	**0.60**	**0.71**	**0.40**	**0.49**	**0.56**
Robinia	0.85	0.86	0.92	**0.25**	**0.24**	0.32
Robiola	**0.65**	**0.94**	0.98	**0.25**	**0.84**	**0.90**
Robot	0.70	0.58	0.72	**0.05**	**0.06**	0.04
Rucola	**0.60**	**0.36**	0.58	**0.45**	**0.20**	**0.31**
Sandokan	0.75	0.70	0.74	**0.25**	**0.06**	0.12
Saroma	**0.70**	**0.62**	0.82	**0.35**	**0.62**	**0.67**
Totò	**0.70**	**0.68**	0.68	**0.10**	**0.52**	**0.56**
Vispo	0.80	0.42	0.52	**0.20**	**0.04**	0.06

For each subject, proportion of choices for 2A in the trials 2A vs. 1A vs. LB, 2A vs. 1A vs. UB, 2A vs. L/2B vs. LB, 2A vs. U/2B vs. UB. Bold data specified within-subject comparisons (the elicitation of the decoy effects between baseline and decoy conditions); the non-bold values referred to the between-subject analyses that verified decoy effects were significantly related to the option architecture (and not to the addition of an extra option). See also main text and Table V (Online Resources) for more details

**Table 2:** Decoy phase, no-Time Pressure condition

Subject	Lower bound (LB)			Upper bound (UB)		
	Baseline	Decoy pro A	Decoy pro B	Baseline	Decoy pro A	Decoy pro B
	2A vs. LB	2A vs. 1A vs. LB	2A vs. L/2B vs. LB	2A vs. UB	2A vs. 1A vs. UB	2A vs. U/2B vs. UB
Cognac	0.90	0.84	0.94	**0.30**	**0.48**	0.50
Gal	0.80	0.86	0.86	**0.20**	**0.22**	0.28
Paprica	0.60	0.40	0.54	**0.00**	**0.04**	0.12
Patè	0.80	0.64	0.92	**0.25**	**0.16**	0.34
Penelope	0.65	0.50	0.44	**0.35**	**0.08**	0.04
Roberta	**0.65**	**0.60**	**0.60**	**0.40**	**0.50**	**0.52**
Robinia	0.85	0.90	0.92	**0.25**	**0.70**	0.64
Robiola	**0.65**	**0.88**	0.96	**0.25**	**0.68**	**0.90**
Robot	0.70	0.62	0.58	**0.05**	**0.06**	0.06
Rucola	**0.60**	**0.32**	0.32	**0.45**	**0.08**	**0.16**
Sandokan	0.75	0.62	0.76	**0.25**	**0.04**	0.06
Saroma	**0.70**	**0.82**	0.92	**0.35**	**0.86**	**0.88**
Totò	**0.70**	**0.92**	0.84	**0.10**	**0.68**	**0.54**
Vispo	0.80	0.66	0.70	**0.20**	**0.10**	0.20

For each subject, proportion of choices for each option in the trials 2A vs. 1A vs. LB, 2A vs. 1A vs. UB, 2A vs. L/2B vs. LB, 2A vs. U/2B vs. UB. Bold data specified within-subject comparisons (the elicitation of the decoy effects between baseline and decoy conditions); the non-bold values referred to the between-subject analyses that verified decoy effects were significantly related to the option architecture (and not to the addition of an extra option). See also main text and Table V (Online Resources) for more details

**Fig. 1:**
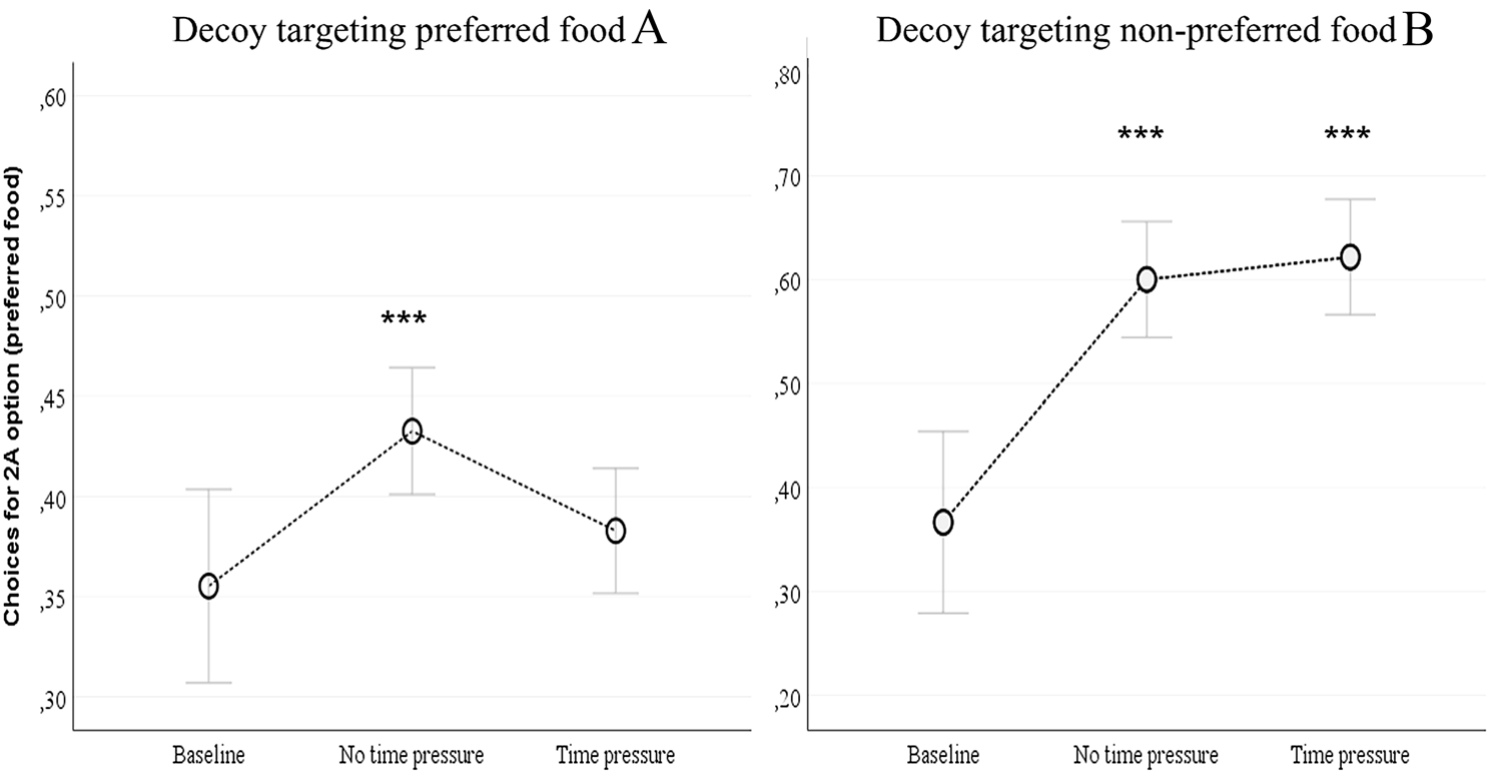
Left panel: Attraction effect. Capuchins’ proportion of choices for the 2A-option as a function of pro-A conditions. ADE was elicited in the condition without time pressure but not in the condition with time pressure. Error bars indicate confidence intervals; ***p < 0.001. Right panel: Repulsion effect. Capuchins’ choices for the 2A-option as a function of pro-B conditions. Decoy additions elicited a repulsion effect regardless of time constraints. Error bars indicate confidence intervals; **p < 0.001

In pro-A trials (*N* = 1885 out of 2800, *N* = 14 subjects), 2A-choices were significantly affected by trial number, within the same session (*z* = 7.82, coeff. = 0.071, *p* < 0.001), whereas they were not significantly affected by session number (*z* = − 0.580, coeff. = − 0.011, *p* = 0.561). This suggests that monkeys increased their preference for the 2A-option across trials within the same session, but this temporary change in preference did not carry on to the following session (i.e., the next experimental day). Moreover, as a control result, there was a significant effect of the bound, with more 2A-choices for lower bound trials than for upper bound trials (*z* = − 6.28, coeff. = − 0.826, *p* < 0.001). More importantly, capuchins performed more 2A-choices in the Decoy phase without time pressure than in the Baseline phase (*z* = 3.12, coeff. = 0.496, *p* = 0.002) and in the Decoy phase with time pressure (*z* = 3.60, coeff. = 0.296, *p* = 0.009), whereas there was no significant difference between the Decoy phase with time pressure and the Baseline phase (*z* = − 1.25, coeff. = − 0.200, *p* = 0.210) (Fig. 1).

As regards pro-B trials, our subject-specific paradigm led to the exclusion of several animals in the Decoy phase, as mentioned above (Table V in the Online Resources): after the exclusion process, we had only six subjects (one in the LB condition and five in the UB condition), inviting caution in interpreting results. Despite these limitations, also in pro-B trials (*N* = 6 subjects), 2A-choices were significantly affected by trial type (decoy/no decoy; *z* = 6.62, coeff. = 0.114, *p* < 0.001) and were not significantly affected by session type (time pressure/no time pressure *z* = − 0.370, coeff. = − 0.013, *p* = 0.710). This result, together with what was observed in pro-A trials, highlights how the presence of a decoy increased subjects’ choices for the 2A-option independently of whether the decoy was targeting A (attraction effect) or B (repulsion effect). Indeed, also in pro-B trials we observed an increase in capuchins’ preference for the 2A-option across trials within the same session. Crucially, capuchins performed more 2A-choices in both the Decoy phases (with and without time pressure) than in the Baseline phase (Decoy with time pressure vs. Baseline: *z* = 5.04, coeff. = 1.517, *p* < 0.001; Decoy without time pressure vs. Baseline: *z* = 5.21, coeff. = 1.570, *p* < 0.001). The bound effect and its interaction were not analysed due to the small number of cases (1 vs. 5 subjects). In short, the most important effect in pro-B trials (6 animals in total) is that the presence of a decoy elicited a repulsion effect (e.g., more choices for the competitor, 2A), and this effect was not significantly modulated by time pressure.

Lastly, we verified if the decoy effect was elicited only when an asymmetrically dominated decoy was present. More specifically, in pro-A lower bound and pro-B upper bound trials, we verified that previously excluded trials (due to the presence of a not dominated decoy) did not yield a pattern of results comparable to the included trials. For these purposes, we ran two control conditional fixed-effects logistic regressions in the decoy pro-A lower bound condition and in the decoy pro-B upper bound condition (the two conditions in which we had to discard the data for 9 out of 14 animals). In each regression, we had two main factors: (i) condition (baseline vs decoy) and (ii) inclusion (excluded vs included trials). If the decoy effect (attraction in pro-A and repulsion in pro-B conditions) was due to the addition of an asymmetrically-dominated decoy, we should have found an interaction effect between condition and inclusion factors. And this was exactly the case. More specifically, in pro-A trials, we found a significant interaction between condition and included trials (*χ*^2^_1_ = 4.30, *p* = 0.038). Subsequent pairwise comparisons verified that attraction effect (that is, an increase in the preference for the 2A-option) only emerged when there was an asymmetrically dominated decoy within the choice set (*z* = − 2.54, coeff. = − 0.552, *p* = 0.011). However, the addition of a not dominated, or double dominated, decoy option did not elicit an increase in target preferences. Symmetrically, the regression on pro-B trials in the upper bound condition highlighted a similar interaction condition*included trials effect (*χ*^2^_1_ = 10.53, *p* = 0.001). Subsequent comparisons revealed an increase in competitor preferences only in the presence of an asymmetrically-dominated decoy (*z* = 5.01, coeff. = 1.567, *p* < 0.001). Conversely, other types of decoys did not shift monkeys’ preferences.

## Discussion

In this study, we observed an attraction effect in capuchin monkeys that were exposed to trinary choices with decoys targeting their favourite food; thus, we successfully replicated previous results obtained with the same species in another colony (Watzek and Brosnan 2020). In addition, we demonstrated the relevance of time pressure in the elicitation of the attraction effect: when capuchin monkeys were imposed a time constraint to make their choice, they were no longer influenced by the decoy options, and they did not show any shift in preferences towards the target alternative. This was presumably because the comparative processes responsible for the attraction effect could not occur. This is in line with well-established results in the literature on human decision makers (Pettibone 2012; Marini and Paglieri 2019), yet it is the first time that a similar finding is reported for capuchin monkeys and, to the best of our knowledge, for non-human animals in general. The implications of this result are potentially far-reaching: insofar as the susceptibility of the attraction effect to temporal constraints is indicative of higher order cognitive processes (i.e., attribute-wise or option-wise comparisons of alternatives, prior to making a choice), observing this phenomenon in capuchin monkeys, a species phylogenetically separated from *Homo sapiens* 35 million years ago, suggests that such comparative processes are evolutionary ancient and invites investigating them also in other species. This is also consistent with the mechanism proposed to underlain the occurrence of decoy effects in amoebas (*Physarum polycephalum*; Latty and Beekman 2011), in which comparative valuation rules were proposed to be at stake. The current study adds to that line of research by finding evidence of comparative decision-making in capuchin monkeys based not simply on their choices, but also on the sensitivity of the attraction effect to time constraints, thus supporting more specific inferences on the underlying cognitive mechanisms.

Another novel result is the observation of a repulsion effect under specific circumstances: whereas this had already been reported in non-human animals, i.e. hummingbirds (Bateson et al. 2002), the authors of that study were inclined to discard it as a methodological glitch. However, the literature on decoy effects on humans does not necessarily support such a dismissive interpretation: repulsion effects have been observed repeatedly with human participants (Aaker 1991; Spektor et al. 2018; Liao et al. 2021; Evans et al. 2021), and several theories have been proposed to explain their appearance, as opposed to attraction effects, based on the decoy-target distance in the attribute space (Liao et al. 2021), aversive associations possibly triggered by very poor decoys (Simonson 2014; Spektor et al. 2018; Kruis et al. 2020), and the order of presentation of the options (Evans et al. 2021). In light of these findings, observing a mixed pattern of attraction and repulsion within the same species should not be automatically discounted as a methodological defect, since it might instead reveal a wider sensitivity of that species to various types of decoy effects.

However, our findings in that regard should be considered exploratory, due to our limited sample size. Nonetheless, two points are worth further consideration. First, whenever a comparison between the impact of pro-A and pro-B decoys was available, we observed the same animals shifting from an attraction effect with pro-A decoys to a repulsion effect with pro-B decoys (in 5 cases out of 6). The fact that the same animals systematically exhibited opposite decoy effects in these two conditions strongly suggests that they were responding to relevant contextual cues, rather than acting randomly. Second, the choice architecture in valid pro-B trials happened to have the characteristics known to facilitate a repulsion effect: while in pro-A trials, the attribute distance between decoy and target was always unitary (1A vs. 2A), in choices with pro-B decoys that distance was twice (2B vs. 4B), thrice (3B vs. 6B), or four times (4B vs. 8B) in magnitude, which, according to Liao and collaborators (2021), would indeed shift preferences away from the intended target of those decoys. While these results remain exploratory, the fact that they are consistent with a theoretical interpretation of the emergence of different types of decoy effects, independently documented in human subjects, suggest to treat them as preliminary evidence of similar processes in capuchin monkeys that are worthy of further exploration, instead of dismissing them as mere noise.

This study also provides useful methodological insights for future experiments. Whereas establishing near-indifference in binary choice, prior to decoy introduction, remains crucial to elicit either attraction or repulsion effects, keeping the decoy/target ratio constant is not the most advisable procedure for decoy construction. Indeed, it may result in numerous invalid trials in trinary choices (e.g., because the decoy ends up being clearly dominated also by the competitor) and it does not allow to systematically explore the impact of target-decoy distance in the attribute space. For instance, animals who had a large upper bound (i.e., 8B, that is the minimum amount of less favourite food they had to be offered to shift their baseline preference towards food B), would allow testing various pro-B decoys with opposite effects: for near-target decoys (e.g., 6B or 7B), we would predict an attraction effect, whereas for far-target decoys (e.g., 4B or lower), we would expect a repulsion effect, like the one we observed in this study. Future studies should take heed of the methodological nuances required to optimally test context effects: this is a topic of much debate also with respect to human subjects (Frederick et al. 2014; Cataldo and Cohen 2019; Kruis et al. 2020; Dumbalska et al. 2020; Spektor et al. 2021), which is made all the more urgent and delicate in light of the many types of contextual influences that affect decision-making across various species (Brosnan and de Waal 2003; Chen et al. 2006; Lakshminarayanan et al. 2008, 2011).

Taken together, our results demonstrate the susceptibility of capuchin monkeys to decoy effects, in ways comparable to those observed in human decision makers: the disappearance of attraction effects under time pressure, in particular, provides a first indication of similar underlying comparative decision processes in, respectively, *Sapajus *spp. and *Homo sapiens*. Our findings also confirm the importance of carefully designing baseline conditions for the successful elicitation of decoy effects, be they attraction or repulsion: previous studies that failed to observe any kind of decoy effect in capuchin monkeys (Cohen and Santos 2017) and other primates (Sanchez-Amaro et al. 2019), in fact, did not preliminary established near-indifference between target and competitor, thus reducing the likelihood of eliciting the effect (Huber et al. 2014). Finally, by preliminarily documenting the variability of context effects, with the same animals reacting in opposite ways to decoys under different choice conditions, this study underlines the usefulness of a greater sophistication in the comparative study of decision biases. Beyond registering the presence of various context effects in non-human animals, it is important to investigate underlying cognitive mechanisms (e.g., focusing more on response times) and systematically map the flexibility of choice patterns, with the aim of developing comprehensive accounts of multiple context effects. This will also help appreciate the ecological relevance of these findings: choice scenarios in which (i) more than two options are available and (ii) one of them is clearly inferior to some but not to other competitors are likely to occur in the natural environment of various species. Imagine for instance a fruit tree in which the fruits differ in ripeness, insect invasion, and ease of access: in such scenarios, the comparative processes responsible for these context effects are likely to play a key role in guiding choice behaviour. Decoy studies offer a useful tool to assess precisely what factors affect comparative decision making in various species, thus offering relevant insights for interpreting their choices not just in laboratory settings, but also in the wild.

## Supplementary Information

Below is the link to the electronic supplementary material.

Supplementary file1 (DOCX 42 kb)
