# Supplementary material for: Context-effect bias in capuchin monkeys (Sapajus spp.): exploring decoy influences in a value-based food choice task
Source: Anim Cogn. 2022 Sep 20;26(2):503–14. doi: 10.1007/s10071-022-01670-0 (PMC9950244; doi:10.1007/s10071-022-01670-0)
Supplement: Supplementary file 1 — Supplementary file1 (DOCX 42 kb) [file 10071_2022_1670_MOESM1_ESM.docx]

# Online Resources

Table I. Preliminary Food Preference phase. Foods used and relative preferences

| **Subject** | **A Food** | **B Food** | **% Preference A Food over B Food** | |
| --- | --- | --- | --- | --- |
|  |  |  | **Session I** | **Session II** |
| **Cognac** | cheerios | raisins | 70% | 75% |
| **Gal** | dried plum | cheerios | 75% | 80% |
| **Paprica** | dried plum | raisins | 70% | 70% |
| **Patè** | pineapple | raisins | 75% | 75% |
| **Penelope** | pineapple | cheerios | 70% | 75% |
| **Roberta** | cheerios | raisins | 70% | 70% |
| **Robinia** | raisins | black olives | 70% | 70% |
| **Robiola** | pineapple | raisins | 70% | 80% |
| **Robot** | raisins | sunflower seed | 75% | 80% |
| **Rucola** | cheerios | raisins | 70% | 70% |
| **Sandokan** | pineapple | raisins | 80% | 75% |
| **Saroma** | pineapple | cheerios | 70% | 75% |
| **Totò** | pineapple | cheerios | 75% | 75% |
| **Vispo** | sunflower seed | raisins | 70% | 70% |

Table II. Pre-test I phase. For each subject, number of sessions needed to reach the criterion and proportion of choices for 2A in the last two sessions

| **Subject** | **Sessions** | **2A vs. 1B** | **2A vs. 8B** |
| --- | --- | --- | --- |
| **Cognac** | 2 | 0.94 | 0.89 |
| **Gal** | 4 | 0.83 | 1 |
| **Paprica** | 3 | 0.94 | 0.89 |
| **Paté** | 2 | 1 | 0.94 |
| **Penelope** | 3 | 0.83 | 0.89 |
| **Roberta** | 3 | 1 | 0.89 |
| **Robinia** | 4 | 1 | 0.89 |
| **Robiola** | 2 | 0.89 | 0.78 |
| **Robot** | 2 | 1 | 1 |
| **Rucola** | 2 | 1 | 0.94 |
| **Sandokan** | 2 | 0.94 | 1 |
| **Saroma** | 2 | 1 | 1 |
| **Totò** | 2 | 1 | 0.83 |
| **Vispo** | 2 | 0.83 | 1 |

Table III. Baseline phase. For each subject, lower bound and upper bound values and proportion of choices for 2A in both trial types (2A vs. LB and 2A vs. UB)

| **Subject** | **Lower Bound** | **Upper Bound** | **2A vs. LB** | **2Avs. UB** |
| --- | --- | --- | --- | --- |
| **Cognac** | 2B | 4B | 0.9 | 0.3 |
| **Gal** | 1B | 4B | 0.8 | 0.25 |
| **Paprica** | 2B | 4B | 0.6 | 0 |
| **Paté** | 2B | 4B | 0.8 | 0.25 |
| **Penelope** | 2B | 4B | 0.65 | 0.35 |
| **Roberta** | 6B | 8B | 0.65 | 0.4 |
| **Robinia** | 2B | 4B | 0.85 | 0.25 |
| **Robiola** | 4B | 8B | 0.65 | 0.25 |
| **Robot** | 2B | 4B | 0.7 | 0.05 |
| **Rucola** | 4B | 6B | 0.6 | 0.45 |
| **Sandokan** | 2B | 4B | 0.75 | 0.25 |
| **Saroma** | 4B | 6B | 0.7 | 0.35 |
| **Totò** | 4B | 6B | 0.7 | 0.1 |
| **Vispo** | 2B | 4B | 0.8 | 0.2 |

Table IV. Pre-test II phase. For each subject, proportion of choices for 2A in the trials 1A vs. 2A, L/2B vs. LB, U/2B vs. UB. All subjects reached the criterion (see main text) in two sessions

| **Subject** | **1A vs. 2A** | **L/2B vs. LB** | **U/2B vs. UB** |
| --- | --- | --- | --- |
| **Cognac** | 1 | 0.89 | 0.94 |
| **Gal** | 0.94 | 0.94 | 0.89 |
| **Paprica** | 0.78 | 0.89 | 0.94 |
| **Paté** | 0.94 | 0.94 | 0.89 |
| **Penelope** | 0.89 | 0.94 | 0.94 |
| **Roberta** | 0.94 | 0.89 | 0.83 |
| **Robinia** | 1 | 0.83 | 1 |
| **Robiola** | 1 | 0.83 | 0.94 |
| **Robot** | 1 | 1 | 1 |
| **Rucola** | 1 | 0.89 | 1 |
| **Sandokan** | 1 | 0.89 | 1 |
| **Saroma** | 0.89 | 0.89 | 0.83 |
| **Totò** | 0.94 | 1 | 1 |
| **Vispo** | 0.94 | 0.94 | 1 |

Table V. Decoy phase. Customized trinary trials and relative exclusion criteria; T = Target; D = Decoy; C = Competitor

|  |  | **Bound** | **Subjects** | **Baseline** | **Decoy condition** | | | **Exclusion criteria** |
| --- | --- | --- | --- | --- | --- | --- | --- | --- |
|  |  |  |  |  | **T** | **D** | **C** |  |
| **Lower**  **bound** | Pro A | 1B | 1 | 2A vs. 1B | 2A | 1A | 1B | C is doubly dominated by T |
|  |  | 2B | 8 | 2A vs. 2B | 2A | 1A | 2B | C is dominated by T on quality |
|  |  | 4B | 4 | 2A vs. 4B | 2A | 1A | 4B | Included |
|  |  | 6B | 1 | 2A vs. 6B | 2A | 1A | 6B | Included |
|  | Pro B | 1B | 1 | 2A vs. 1B | 1B | 0.5B | 2A | T is doubly dominated by C |
|  |  | 2B | 8 | 2A vs. 2B | 2B | 1B | 2A | D is dominated by T and doubly dominated by C |
|  |  | 4B | 4 | 2A vs. 4B | 4B | 2B | 2A | D is dominated by both C (on quality) and T (on quantity) |
|  |  | 6B | 1 | 2A vs. 6B | 6B | 3B | 2A | Included |
| **Upper**  **bound** | Pro A | 4B | 8 | 2A vs. 4B | 2A | 1A | 4B | Included |
|  |  | 6B | 3 | 2A vs. 6B | 2A | 1A | 6B | Included |
|  |  | 8B | 2 | 2A vs. 8B | 2A | 1A | 8B | Included |
|  | Pro B | 4B | 8 | 2A vs. 4B | 4B | 2B | 2A | D is dominated by both C (on quality) and T (on quantity) |
|  |  | 6B | 3 | 2A vs. 6B | 6B | 3B | 2A | Included |
|  |  | 8B | 2 | 2A vs. 8B | 8B | 4B | 2A | Included |

Table VI. Number of subjects and trials used in within-subject analyses across experimental conditions.

|  |  | **Animals** | **Baseline** | **Decoy** | **Decoy TP** |
| --- | --- | --- | --- | --- | --- |
|  |  |  | Trials | | |
| **Lower bound** | Pro A | 5 | 100 | 250 | 250 |
|  | Pro B | 1 | 20 | 50 | 50 |
|  |  |  |  |  |  |
| **Upper bound** | Pro A | 14 | 280 | 700 | 700 |
|  | Pro B | 5 | 100 | 250 | 250 |
